# The Medicinal Phage—Regulatory Roadmap for Phage Therapy under EU Pharmaceutical Legislation

**DOI:** 10.3390/v16030443

**Published:** 2024-03-12

**Authors:** Timo Faltus

**Affiliations:** Chair of Public Law, Law School, Faculty of Law, Economics and Business, Martin-Luther-University Halle-Wittenberg, 06099 Halle an der Saale, Germany; timo.faltus@jura.uni-halle.de

**Keywords:** bacteriophage (phage), phage therapy, antimicrobial resistance, regulatory framework, personalised medicine, magistral formula, designer phages, advanced therapy medicinal products (ATMP)

## Abstract

Bacteriophage therapy is a promising approach to treating bacterial infections. Research and development of bacteriophage therapy is intensifying due to the increase in antibiotic resistance and the faltering development of new antibiotics. Bacteriophage therapy uses bacteriophages (phages), i.e., prokaryotic viruses, to specifically target and kill pathogenic bacteria. The legal handling of this type of therapy raises several questions. These include whether phage therapeutics belong to a specially regulated class of medicinal products, and which legal framework should be followed with regard to the various technical ways in which phage therapeutics can be manufactured and administered. The article shows to which class of medicinal products phage therapeutics from wild type phages and from genetically modified (designer) phages do or do not belong. Furthermore, the article explains which legal framework is relevant for the manufacture and administration of phage therapeutics, which are manufactured in advance in a uniform, patient-independent manner, and for tailor-made patient-specific phage therapeutics. For the systematically coherent, successful translation of phage therapy, the article considers pharmaceutical law and related legal areas, such as genetic engineering law. Finally, the article shows how the planned legislative revisions of Directive 2001/83/EC and Regulation (EC) No 726/2004 may affect the legal future of phage therapy.

## 1. Introduction

Bacteriophages (phages) are viruses that, in view of the worsening antibiotic crisis, are intended to be used as a standard treatment for bacterial infections in the future [1,2,3,4,5,6]. The regulatory handling of phage-based medicinal products and the legal structure of the corresponding translational pathways, from basic research to routine use, have not yet been conclusively clarified under EU pharmaceutical law. Unanswered legal questions can hamper practical medical translation, since legal feasibility is not assured from the outset. Considering the technical requirements of phage therapy, the legal roadmap described here aims to serve as a basis for an interdisciplinary discussion between scientists and physicians on the one hand, as well as lawyers and regulators for the successful legal translation of phage therapy on the other hand.

Regarding the application of phages to humans, there are—in contrast to veterinary medicine—currently no phage-specific regulations on the level of EU law. So far, the European Medicines Agency (EMA) has only stated that some principles of the “Guideline on the evaluation of medicinal products indicated for treatment of bacterial infections” can also be applied to phages [7], after the EMA explicitly excluded this in the previous version of this guideline [8]. However, for about 15 years, successful individual cases have been documented and clinical studies have been conducted [4,9,10,11,12,13,14,15,16,17,18].

The regulatory handling of phages is based on the opposite understanding of the previous handling of human viruses in medicine. Up till now, viral infections have been diseases to be treated. Tissues, organs, and blood to be transferred are examined for the absence of viruses, and, at most, attenuated viruses are used as vaccines or as vehicles (vectors) in gene therapy [19].

The importance of finding new antibacterial substances is illustrated by the calculations published for 2019 of approximately 1.27 million deaths worldwide each year due to antibiotic-resistant bacteria [20]. If this development is not stemmed by new pharmaceutical products—albeit according to critically revised calculations [21]—up to 10 million deaths per year could be caused by antibiotic-resistant bacteria by 2050 [22]. For the EU and European Economic Area, about 33,000 deaths due to antibiotic-resistant bacteria were already estimated annually in 2015 [23]. These figures are the deaths directly attributable to the resistances. The numbers of deaths where resistance is an additional factor are significantly higher.

To date, there is—to the best of our knowledge—no phage-based medicinal product for human use approved under EU law. Only in Slovakia and the Czech Republic is a phage therapy medicinal product on the market, Stafal^®^ [24,25]. However, this product entered the market of these countries before they both joined the EU, so this therapeutic has not been assessed under EU law.

Where phage therapeutics are currently used in the EU, this is usually done as an individual experimental therapy attempt or as part of a clinical trial. In these cases, phage preparations individually prepared on site are applied or single imports are used, especially from non-European countries such as Georgia, where phage therapy has been developed and applied for about 100 years [26,27,28]. In the EU, Poland [29,30] and Belgium [27,31,32,33] have taken the lead in the development of phage therapy. Also in France [34] and in Germany, research is being conducted to translate phage therapy into a standard treatment for bacterial infections; in Germany, with funding from the EU [35], the German Federal Ministry of Education and Research (BMBF) [36], and the G-BA (German Federal Joint Committee of physicians, dentists, hospitals, and health insurance funds) [37].

Given the progress in the medical and technical development of phage therapy, in June 2015, the EMA held a workshop on the therapeutic use of phages in human medicine [38]. For the EU pharmaceutical legislation, regulatory assessments have been published, mostly from the medical and pharmaceutical research community [3,39,40,41,42]. In the meantime, in the area of the European Pharmacopoeia, a consultation process is ongoing for the creation of a general chapter on phage therapy active substances [43], and in the EU Commission’s proposal for a new Directive for medicinal products for human use, presented in April 2023, phages are mentioned as medicinal products for human use [44]. Finally, in December 2023, the EMA launched a process to prepare a concept paper on the development and manufacture of phage medicinal products for human use [45]. Also in the USA, an interdisciplinary discourse on regulatory issues relating to phage therapy is taking place [46].

Building on this previous work, the paper describes from a jurisprudential view the legal and regulatory aspects for the translation and regulation of phage therapy under EU law, and under what circumstances and to what extent the law of the individual EU Member States applies. Due to its comprehensive coverage, the article has a modular structure so that the various legal issues can be accessed either individually or consecutively. To access only individual legal issues, it is possible to start with Table 1 and Table 2, with links to the respective chapters on the specific legal issues.

## 2. What Are Phages?

### 2.1. Structure and Occurrence of Phages

Phages are viruses that infect bacteria, but not human cells [47,48]. Even though phages have different phenotypes, the most memorable (simplified and enlarged) picture of how to imagine a medically relevant (tailed) phage is with a picture of an Apollo lunar module. In the head part of the phage (capsid, made of proteins) is the nucleic acid, which consists of DNA. This is followed by the tubular tail, at the lower end of which there is a tooth-like spike for penetrating the bacterial membrane. Around the spike are tail fibres [49], figuratively comparable to the stilt-like landing legs on the lunar module.

The lack of awareness of phages outside of scientific communities is inversely proportional to their ubiquitous occurrence worldwide. Phages are everywhere where there are bacteria. Phages were found in amounts of up to 250 million phages per millilitre in surface water, and up to 15 million could be found in marine water [50,51]. In one gram of certain soil types, phages are estimated to exist in quantities of more than one billion [52,53] without this being visible to the human eye. In addition, phages regularly reside on human skin [54,55] and in other parts of the body [56]. In a healthy person, about 31 billion phages can enter the body every day from the gut, where they support microbial homeostasis [57]. This natural phage spreading has no harmful effects, but it is also not therapeutically usable to fight bacterial infections.

Like all viruses, phages cannot reproduce independently but require host organisms for this purpose. In the case of phages, these host organisms are bacteria. Phages are host-specific, i.e., a particular species of phage can usually only infect a particular type of bacteria. Therefore, depending on the phage species, human pathogenic bacteria can be the host organism [47,48,58].

### 2.2. Infection Behaviour and Pharmacological Mode of Action

The therapeutic effect of phage therapy is based on the selective use of certain phage species to kill only pathogenic bacteria, thereby treating the infectious disease. Phages can be used in such a way that they are not only harmless to humans but also harmless to beneficial bacteria of the gastrointestinal flora. As a result, phages have a more specific effect than antibiotics. Depending on the infection, either a single phage type or phage mixtures (so-called phage cocktails) can be used to achieve the required efficacy spectrum in cases of infection with different bacterial species [26,33,47,59,60]. The focus of these efforts to date has been on the utilisation of naturally occurring phages, so-called wild type phages. In addition, studies are being carried out on the use of genetically modified phages (also known as designer phages or synthetic phages), which, compared to wild type phages, for example, are expected to show a broader host spectrum or more rapid multiplication [46,58,60,61,62,63,64,65].

Regarding the application, it should be kept in mind that a therapy-relevant quantity of phages is required, i.e., the number of phages administered in the specific case (a different medicinal/technical question is whether this number refers to the same phage type or to different phage types). This quantity is typically manufactured by a phage bank offering this service in addition to the storage of phages or by a facility/firm specialised in the amplification of phages. In this process, suitable host bacteria are typically used to amplify the phages [66,67,68], although cell-free (i.e., bacteria-free) approaches are now also being developed to amplify phages [69]. The practical relevance of the therapeutically required quantity of phages should not be underestimated, since phages are also recognised and sometimes inactivated by the human immune system. However, if there are enough suitable host cells, the phages may be able to multiply faster than the immune system can destroy the phages [70,71,72,73,74].

For medical use, the virulent/lytic phages are of particular interest [1,75]. These phages recognize their host by means of its surface structures (receptors), whereby the tail fibres of the phage and the receptors must fit like a lock and key, since only then can both link to each other (adsorption). Subsequently, the phages induce their host cell to produce new phages by introducing nucleic acids into the host, which genetically reprograms the host. The bacteria infected by the phages then produce further phages, which are released by the bacteria “instrumentalised” in this way, destroying the bacterium under lysis (=disintegration) [58,76,77,78]. This cycle repeats continuously, whereby a status can be reached at which all hosts are destroyed at a given location. If the then still existing phages do not find any further hosts, they decay and/or are carried by external influences (environmental, in the human body by the reticulo-endothelial system) to another location with further host bacteria [1,26,47,74,79,80] since phages cannot actively move themselves [78].

### 2.3. Do Phages Have a Pharmacological Action in the Legal Sense?

Pharmaceutical legislation distinguishes between medicinal products by virtue of their presentation and medicinal products by virtue of their function. This distinction has significance (e.g., in the event of disputes between developers/suppliers and authorities) for legal issues relating to the demarcation of medicinal products from other products such as medical devices, biocides, cosmetics, and food, as well as for questions of liability and advertising.

One of the legal criteria of a medicinal product by virtue of its function is that the product in question has a pharmacological, immunological, or metabolic effect according to Article 1(2)(b) of Directive 2001/83/EC. For the legal assessment of phages, it is therefore necessary to clarify whether the scientific mode of action of phages is within the meaning of Article 1(2)(b) of Directive 2001/83/EC. If this mechanism of action cannot be demonstrated, the therapeutic product may be a medicinal product by virtue of its presentation within the meaning of Article 1(2)(a) of Directive 2001/83/EC, provided that the substance has properties for the treatment or prevention of human disease.

At first glance, for the legal determination of the mechanism of action of phage therapeutics, phage therapeutics can be compared with classic (chemical-based, chemicals) antibiotics. Both therapeutic agents have the same target, namely the killing of pathogenic bacteria. However, EU pharmaceutical law does not explicitly define the mechanism of action of classic antibiotics, and the European Court of Justice (CJEU)—as far as can be seen from the databases—has not yet explicitly dealt with questions of how the effect of classic antibiotics should be legally classified. In the proceedings on the question of the extent to which the antibacterial mode of action of chlorhexidine in mouthwash solutions is to be legally assessed as a pharmacological mode of action, the CJEU did not legally define the mode of action of chlorhexidine but only determined the standard of interpretation for answering this question by the Member State courts. This lack of legal discussion of the mechanism of action for classic antibiotics at the EU legal level does not mean that the characterisation of classic antibiotics as medicinal products is legally unclear or controversial; it only says that their mechanism of action has not yet been disputed at the EU level. This lack of judicial discussion is also not to be confused with the question of what is to be legally understood as a pharmacological effect in general and specifically in regard to antibiotic substances. Currently, the CJEU has to answer the question of whether it is a pharmacological effect in legal terms if D-mannose reversibly binds to bacteria through hydrogen bonds and thereby prevents the bacteria from binding to human cells (Case C-589/23). In any case, since phages are obviously structurally/chemically different from classic antibiotics despite their similar use, and since the mechanism of action of classic antibiotics has not yet been determined by an EU court, an independent legal assessment of the mechanism of action must be carried out for phage-based therapeutics. Although, no controversial administrative proceedings or court cases on the legal classification of phage therapeutics have come to public attention to date. As the field is just developing, this is not surprising. Nevertheless, the interdisciplinary phage therapy community can and should anticipate the legal arguments for the regulatory handling of phage therapeutics in the interest of a proactive technology assessment to promote smooth translation.

In any case, the mode of action of phages to treat bacterial infections is not immunological since the therapeutic effect of phages is not mediated by the immune system.

However, the effect of phages is pharmacological, although (a) the effect of phages is not on human cells but on the pathogenic bacteria that have infected humans, and (b) the pharmacological effect is not the interaction of a low molecular weight chemical with a receptor, as has been the case so far, but the interaction of a virus with a receptor.

A characteristic of a pharmacological effect is the interaction between the substance in question and the cell structures of the applicant, in particular receptors, whereby this interaction results in a direct reaction or blocks the reaction of another agent [81]. Typically, this interaction goes beyond the effects of nutrients in distinction to food [82]. The interaction between phages and the matching receptors of bacteria corresponds to this mechanism of action. However, on closer inspection, the pharmacological effect is in the phage-bacteria ratio and not in the phage-human ratio. In contrast, antiviral medicinal products act directly in the pharmaceutical–human relationship, since, for example, the docking of the virus to a human cell or the replication of the virus in the cell is to be prevented.

However, the CJEU has already clarified that a pharmacological effect cannot only be assumed if there is an interaction between the molecules of the substance in question and a cellular component of the user’s body, but that an interaction between this substance and any cellular component present in the user’s body, such as bacteria, viruses, or parasites, is sufficient [83]. Thus, it can be assumed that phage therapeutics have a pharmacological effect within the meaning of pharmaceutical law.

Even though the deduction of the pharmacological effect has been discussed and affirmed so far in the relationship between chemical, low-molecular agents and the target structure, this also applies to the effect of phages, which are more complex and higher-molecular than the aforementioned chemicals, since the same key/lock principle is involved and also no reasons are apparent that would justify a limitation to a certain molecular size for this mode of action.

In addition to the pharmacological effect—as also stated by the CJEU—the pharmacological substance has to significantly restore, correct, or influence the physiological functions of humans when used as intended [84]. This can be affirmed in the case of phage therapeutics because, as a result of the phage-mediated elimination of bacteria, healing processes such as wound closure or the reduction in inflammation at infected joint replacements, for example, are significantly influenced as physiological functions.

Whether the effect of phages in the legal sense is also metabolic does not need to be discussed further since the pharmacological effect has already been identified and, due to the naming of alternatives, it is no longer relevant whether the effect is also metabolic and/or immunological.

## 3. Application Settings of Phage Therapy

### 3.1. One-Fits-All in Advance vs. Patient-Specific on Demand

Phage therapy can be realised in two settings [33,42,59,85,86,87]. On the one hand, the approach is comparable to medicinal products manufactured in advance, independently of the patient. In this context, uniform phage mixtures are manufactured on an industrial scale, assembled in such a way that the mixtures are immediately available when needed [88]. The mixture is composed in such a way that the cocktail contains at least one phage type that kills the pathogenic bacterial species occurring in the specific case.

On the other hand, research is being conducted to realise individualised phage therapy [89,90,91]. This can be carried out in a physician’s practice using physician-made phage therapeutics, in hospitals using phage medicinal products prepared in the hospital pharmacy, or by a commercial pharmaceutical entrepreneur who supplies the hospital as a third-party provider. Similarly, a physician may use such commercially prepared but individual phage therapeutics in his or her medical practice.

What all variants of individualised phage therapy have in common, in contrast to the approach of an industry pre-made uniform medicinal product, is that the patient-specific therapeutic product is prepared only after diagnosis and based on the bacterium or bacteria to be targeted. A phagogram is used to determine which phages correspond therapeutically to the bacteria previously isolated from the patient. The phagogram, similar to an antibiogram for antibiotics, is used to test the sensitivity of the bacterial isolate to previously appropriately characterised phages [3,31,42,92,93,94,95].

Since different phages are required for phage treatment depending on the infection, a phage bank is necessary in which the relevant phages are kept in stock and from which phages are then taken and amplified. Especially in individualised cases, the spatial and organisational proximity of the patient, microbiological analysis, phage bank, and preparation is necessary to enable prompt treatment [80,95].

The patient-specific path is pursued because, on the one hand, bacterial infections differ individually due to the numerous subtypes/strains of bacterial species that are already numerous by themselves and, on the other hand, because phages can be subtype-specific. Capturing this diversity in a “one-fits-all” pre-made medicinal product while maintaining sufficient phage concentrations is so far more difficult to implement than the patient-specific approach [93,96]. Irrespective of this, the use of prefabricated phage-containing therapeutics is recommended for the therapeutic use of certain polyvalent phages, as these phages can capture a corresponding number of strains of a bacterial species [3,9,97,98].

### 3.2. Interaction between Phage Bank and Medicinal Product Manufacturers

Given the technical challenges and the need to ensure the quality of phage therapeutics, regarding the patient-specific approach, it is unlikely that phage therapeutics will be prepared in an average physician’s practice or in a community (neighbourhood) pharmacy. This is because the culture of phages in a phage bank and the amplification of phages are not activities that a community pharmacy or a doctor’s practice can manage in addition to all their other activities. Rather, it is expected that dedicated phage banks will be established—as can be observed to some extent today [3,93,99]—that can provide the collection and characterisation of therapeutically required phages. Either these phage banks or associated facilities can then amplify the phages in the quantity and quality required for treatment on demand. Since the preparation of the ready-to-use phage medicinal product, particularly with respect to the dosage (galenic) form, in turn requires specialised pharmaceutical knowledge not typically associated with a phage bank or a facility for the amplification of phages, there are two main scenarios to consider for the preparation of the ready-to-use patient-specific phage therapeutics: On the one hand, phage therapeutics can be prepared in hospital pharmacies by obtaining phages from a (commercial) phage bank. On the other hand, the preparation can be carried out outside hospital pharmacies by appropriately specialised commercial manufacturers, who then deliver it to the hospital pharmacy for further distribution within the hospital.

The extent to which manufacturers who, irrespective of the individual case, only manufacture certain predefined static phage therapeutics (cocktails) on an industrial scale, particularly on the basis of polyvalent phages, keep the required phages in stock and multiply them in their own in-house phage bank, or are supplied with phages by a third-party provider, is primarily an organisational, entrepreneurial decision that must subsequently be taken into account from a regulatory perspective.

## 4. What Are Phages under Pharmaceutical Regulation and What Are They Not?

### 4.1. Are Phages Pharmaceutical Substances?

According to Article 1(3) of Directive 2001/83/EC, viruses are not mentioned explicitly in the definition of the term “substance”.

If the Commission’s proposal [44] should enter into force in its current version, viruses would be explicitly regulated as a substance within the scope of microorganisms, Article 4(1)(2)(e). However, since viruses, and thus phages, have so far not been literally addressed by Directive 2001/83/EC, it must be determined whether viruses can possibly be classified under the current substance concept by way of interpretation. The clarification of this question is of importance since the nature of the substance is the central requirement for a product to be an active substance and/or medicinal product.

The substance definition of Directive 2001/83/EC states that substances are all substances of any origin. However, this is followed by a categorisation into substances of human, animal, plant, and chemical origin.

In any case, phages are not of chemical origin [100], since phages are not chemical elements, natural chemical substances, or chemical compounds obtained by processing or synthetic means. In addition, phages are not substances of human origin, as phages are not human blood, products derived from it, or other products of the human body. In the case of substances of animal and plant origin, Article 1(3) of Directive 2001/83/EC mentions, among others, secretions, toxins, or substances obtained by extraction. Phages also do not fulfil these requirements.

In addition, Article 1(3) of Directive 2001/83/EC mentions microorganisms. However, since phages as viruses are not living organisms from a scientific point of view, it is questionable whether viruses can be microorganisms under the wording of the law. Phages are excreted from the bacteria they have infected or are collected in nature. These variants are not included in the substance definition of Article 1(3) of Directive 2001/83/EC. This suggests that viruses cannot be substances under pharmaceutical law and thus cannot be medicinal products in the sense of Directive 2001/83/EC.

However, the legal substance nature of viruses in general and phages in particular can be concluded by systematic legal interpretation of the EU pharmaceutical law.

On the one hand, within Directive 2001/83/EC, for example, Annex I, Part IV, 3.2.1.3 describes medicinal products containing viruses or viral vectors. Viruses, and thus also phages, must therefore have a substance nature, since the definition of medicinal products is based on the substance nature.

On the other hand, in Article 2(1) of Directive 2001/18/EC “organism” refers to any biological entity capable of reproducing or transferring genetic material. By Annex I A, Part 1, 1. it becomes clear that this concept of organism legally also includes viruses, since the Annex explicitly mentions viruses that (as an organism) can be genetically modified and can transfer genetic material. In the same way, the legal status of an organism also results from Directive 2009/41/EC. According to Article 2(a) of Directive 2009/41/EC, the term “microorganism” refers, among others, to viruses. Thus, the EU legislator has demonstrated that viruses are at least not excluded from the outset, per se, from the legal concept of the microorganism. Since viruses and thus also phages can be microorganisms according to EU law, they can also be microorganisms within the meaning of the term microorganism in Article 1(3) of Directive 2001/83/EC and thus have substance status under medicinal product legislation (cf. Table 1).

### 4.2. Active Substance and/or Medicinal Product and Its Dosage Form

Due to the legal substance nature of phages, phages or phage-containing therapeutics can be (a) an active substance according to Article 1(3a) of Directive 2001/83/EC and/or (b) a medicinal product according to Article 1(2) of Directive 2001/83/EC. The classification of phages or phage-containing therapeutics in these legal terms cannot remain undecided since the legal provisions for active substances and medicinal products are not identical.

First, however, it must be determined whether, from a legal point of view, the therapeutic effect, i.e., the bactericidal effect, originates from the phage as such or only from the nucleic acid that the phage injects into the target bacterium. Phages are only functional in their entirety to infect bacteria. The phage nucleic acid by itself cannot inject itself into a bacterium to initialise the bactericidal effect. Therefore, phages used therapeutically are to be considered in their entirety of nucleic acid and capsid for the assessment of their properties as active substances and/or medicinal products.

Even if the pharmaceutical effect originates from the phage, a phage medicinal product cannot contain plain “naked” phages. The phages would be therapeutically non-handleable in this undosed form. Instead, depending on the infection [101], the phages need to be prepared with additional excipients (Article 1(3b) of Directive 2001/83/EC) into the required dosage form. Within a medicinal product, phages are thus active substances within the meaning of Article 1(3a) of Directive 2001/83/EC, whereby an active substance is “*any substance or mixture of substances intended to be used in the manufacture of a medicinal product and that, when used in its production, becomes an active ingredient of that product intended to exert a pharmacological, immunological or metabolic action […].”*

Only the final dosage form, in the case of patient-independent manufacture in advance outside pharmacies, is a phage medicinal product regulated by Directive 2001/83/EC (Article 2(1), *e contrario* Article 3 of Directive 2001/83/EC) or, in the case of patient-individual preparation in pharmacies, a phage medicinal product not regulated by Directive 2001/83/EC (Article 3 of Directive 2001/83/EC) but regulated under the medicinal product legislation of the Member States (cf. Table 1 and Table 2).

The dosage form of the phage-containing medicinal product depends, among other things, on the infection site (internal, external, local, or systemic). Possible dosage forms under investigation include the incorporation of phages into gels, ointments, gauze, or liquid solutions for injection [14,77,96,101,102,103,104,105]. In addition, inhalable phage therapeutics are being developed for the treatment of respiratory diseases [71,106,107].

The dosage form itself can have an impact on the legal handling if, for example, the dosage form consists of a medicinal product component and a medical device component. In this case, it is necessary to clarify to what extent the regulatory framework of medicinal products and/or medical devices is relevant for the product as a whole (Section 4.8).

### 4.3. Not Cells or Tissues

So far, the literature has primarily described (positively) to which class of medicinal product phages may belong [1,3,108]. However, such an investigation is not complete. For an unambiguous classification, it must also be (negatively) determined which classes of medicinal products are not relevant. The classification is legally unambiguous only if only one class of medicinal product is relevant for that therapeutic product. If from the outset only one class of medicinal product is investigated and justified, another class of medicinal product may still be relevant. If this were the case, questions of regulatory demarcation would have to be answered.

Phage therapeutics are neither cells according to Article 3(a) nor tissues according to Article 3(b) of Directive 2004/23/EC. These definitions refer to cells or compounds of cells as living (metabolically active) entities. Phages as viruses do not fulfil this requirement. There is also no legal fiction to equate therapeutically used viruses with cells or tissues. For the same reasons, the planned “EU Regulation on substances of human origin” (SoHO Regulation) [109] for the further harmonisation of the rules for blood and tissue donations has no relevance for the regulation of phage therapy.

### 4.4. Not Blood, Not Blood Products

Phage therapeutics are not blood products since phages are not derived from plasma blood, according to Article 3(c) of Directive 2002/98/EC.

### 4.5. Not Xenogeneic Medicinal Products

Phage therapeutics are not xenogeneic medicinal products because phages are not animals. The legal requirement for xenogeneic medicinal products is that the medicinal product in question consists of or contains animal tissues or cells. This follows from “Annex I, Part IV, 3.3.2.1. Starting materials, (d)” of Directive 2001/83/EG, which states that for xenogeneic cell-based products, information on the source of animals shall be provided.

### 4.6. Not Immunological Medicinal Products (Vaccines)

With respect to use for the treatment of bacterial infections, phage therapeutics do not legally qualify as immunological medicinal products (here, vaccines) under Directive 2001/83/EC. Although phage therapeutics are used to treat infectious diseases caused by bacteria, not every treatment for an infectious disease is a vaccination. Similarly, the treatment of a bacterial infection by means of a chemical antibiotic is not a vaccination. Rather, for the concept of a vaccine within the meaning of Article 1(4) of Directive 2001/83/EC, it is necessary that an active or passive immunity is induced. However, phages do not cause immunity; the bactericidal effect of phages is not mediated by the human immune system.

Since the mode of action of phage therapy is not mediated by the immune system, phage therapy does not provide immunity to a reoccurring infection, either with the same bacterium or with a different bacterium. In cases of reinfection, a new (phage) treatment is required. This is also a difference from the use of viruses or virus components in vaccinations. In vaccinations, inactivated viruses or viral components are applied to induce the immune system to produce antibodies to prevent the manifestation of the disease associated with the virus upon contact with the active virus. However, phage therapy against bacterial infections is not preventive, but curative. A different legal assessment applies to phage-based therapeutics that are intended to induce immunity via phage display; these are immunological medicinal products (vaccines), Section 4.9.1 and Table 1).

### 4.7. Not Biocides

Phages used therapeutically are not biocides as defined by Regulation (EU) No. 528/2012. In the sense of Article 3(1)(a) of this Regulation, phages can destroy harmful organisms by means other than mere physical or mechanical action and thus have a biocidal effect. However, phages do not fulfil the substance quality required by Article 3(1)(a) of Regulation (EU) No. 528/2012. According to Article 3(2)(a) of Regulation (EU) No. 528/2012, in conjunction with Article 3(1) of Regulation (EC) No. 1907/2006, only chemical substances and their compounds are classified as substances. However, viruses are not mere chemical substances and have a substantially different structure at the molecular level than chemical elements and their compounds.

### 4.8. Not Medical Devices

Phage therapeutics are not medical devices because, according to Article 1(6)(h) of Regulation (EU) No. 2017/745, the Medical Devices Regulation does not apply to products that consist of or contain viruses to achieve or support the intended purpose of the product. Another (regulatory) question is whether items used for the application of phage therapeutics, such as pharmacologically, metabolically, and/or immunologically inert carrier items (gauze, gel, etc.), are medical devices. If such carrier items are to be legally classified as medical devices, the overall therapeutic product may represent a combination product of a medical device component and a medicinal product component. However, since the intended main effect (namely the antibacterial effect) of such a combination product also results from the phage (=virus) component, such a combination product as a whole cannot be a medical device. For such combination products—as for other combination products—it is then necessary to clarify on a case-by-case basis how medical device components and medicinal product components interact with each other (cf. Article 1(8), (9) of Regulation (EU) No. 2017/745) to determine the relevant regulatory framework.

The questions of medical device regulation, which regulation applies to the diagnostics (devices, chemicals) for determining the phages to be used in individual cases, must be clarified independently (and are not discussed here).

### 4.9. Genetically Modified (Micro-) Organism

#### 4.9.1. Wild Type Phages and Genetically Modified Phages

A naturally occurring phage (wild type) is neither a genetically modified organism (GMO) according to Article 2(2) of Directive 2001/18/EC nor a genetically modified microorganism (GMM) according to Article 2(b) of Directive 2009/41/EC. Although a phage as a virus is an organism in terms of Article 2(1) of Directive 2001/18/EC or a microorganism in terms of Article 2(a) of Directive 2009/41/EC, the required modification of the genetic material is missing. For the therapeutic use of wild type phages, genetic engineering law is therefore neither applicable directly nor by reference from Directive 2001/83/EC, e.g., from Annex I, Part I, 1.6 of Directive 2001/83/EC.

However, as shown, there is also research on the use of genetically modified phages. Whether these genetically modified phages are GMOs or GMMs within the meaning of genetic engineering law must be decided based on genetic engineering law. This finding should not be confused with the question of whether the therapeutic use of genetically modified phages is a gene therapy medicinal product (GTMP) under pharmaceutical law, as there is no statutory automatism to link and uniformly evaluate these questions. If genetically modified phages are used therapeutically, the question of whether they are GTMPs needs to be answered independently. For this purpose, it is first necessary to clarify for what the genetically modified phage is used, since the different uses have different legal frameworks.

On the one hand, like wild type phages, genetically modified phages can be used to treat bacterial infections. In contrast to wild type phages, for example, genetically modified phages are intended to have a therapeutically improved host spectrum. The extent to which this is legally a gene therapy must be clarified independently (Section 4.10).

On the other hand, genetically modified phages can be used for vaccination. However, since phages do not infect human cells, phages used for vaccination are to be genetically modified in such a way that they display proteins (antigens) on their surface (so-called phage display), which are recognised by the human immune system [110]. Subsequently, the immune system generates matching antibodies without a disease-causing infection, as is usually the case with vaccinations. These approaches have been investigated, for example, in the search for COVID-19 vaccines [111]. In this use, genetically modified phages are not a GTMP, since according to Annex I, part IV, 2.1, sentence 4 of Directive 2001/83/EC, vaccines against infectious diseases are not GTMPs, even if GMOs in the sense of genetic engineering law are used. This regulatory classification is not identical with the question of the extent to which an environmental risk assessment may be necessary for therapeutics containing GMOs (cf. Annex I, Part I, 1.6 of Directive 2001/83/EC).

#### 4.9.2. (No) CRISPR Specifics?

Regarding genetically modified phages, there is (in principle) no need for a specific discussion under pharmaceutical law on the use of genome editing methods such as the CRISPR/Cas method. The legal discussion on genome editing concerns questions of genetic engineering law. This discussion was motivated by the fact that genome editing methods can, among other things, directly modify genetic material in a way that can be achieved under natural conditions by undirected mutagenesis, or by the traditional methods of undirected mutagenesis using radiation or chemical influence, whereby these traditional methods do not result in a legally regulated GMO in the sense of Directive 2001/18/EC. The question in genetic engineering law was therefore whether the direct methods of genome editing (i.e., only those corresponding to a mutation) should be treated legally like the traditional, undirected methods. The CJEU has rejected this, saying that genome-editing-caused mutations lead to regulated GMOs [112]. However, genome editing methods can also be used to generate recombinant DNA, i.e., to modify larger DNA segments (larger than mutation). In this application, even before the ruling of the CJEU, it was legally undisputed that, from a legal point of view, the result is a recombinant nucleic acid and thus a legally regulated GMO.

Admittedly, the discussion on the legal handling of the various uses of genome editing methods in genetic engineering law has no legally binding influence on the definition of the GTMP because the definition of the GTMP is not based on the legal definition of the GMO according to the genetic engineering law but is legally independent (see above Section 4.9.1), whereby the presence of a recombinant nucleic acid is particularly important for the GTMP. However, the different ways of using genome editing methods have an impact on the legal classification of genetically modified medicinal phages. If a medicinal phage containing recombinant DNA (or RNA) is generated by genome editing or any other method, then it is a GTMP, provided that the other requirements of the legal definition of GTMP are also met (Section 4.10). The same is true if phages are used to deliver the CRISPR/Cas system to bacteria in the human body. In this approach, the bactericidal effect should be achieved not only by phage-mediated lysis but additionally by CRISPR/Cas-mediated genetic modification of the target bacteria [113]. In terms of pharmaceutical law, these are also GM-phages because their nucleic acid has been recombined to transfer the CRISPR/Cas system.

However, if the nucleic acid of a medicinal phage were to be modified by mutagenesis (whether by irradiation, chemically, genome editing, etc.), the result would not be a recombinant nucleic acid, because recombinant in the current technical and legal understanding of the term presupposes that other nucleobases, in particular from another organism, are artificially inserted into an existing nucleic acid molecule, i.e., recombined. However, mutagenesis does not involve recombination but rather the exchange of individual base pairs within the organism without the incorporation of foreign DNA/RNA (from another organism) into the nucleic acid molecule under consideration. As a consequence, the legal requirements of the definition of a GTMP would not be fulfilled since this definition is based on a recombined nucleic acid. Whether the definition of a GTMP under the proposed reform of Directive 2001/83/EC [44] will change this assessment will need to be clarified, since in the future sequence-specific edits (i.e., not necessarily recombinations of different nucleic acids) of the target genome should also be able to constitute a GTMP.

#### 4.9.3. Phages from Phage Training

Since the methods of phage training are also based on mutations of the phage genome [114,115,116], this suggests that the (mutated) phages generated by phage training should legally not be classified as GTMP. Instead, phages from phage training are to be treated legally as wild type phages.

#### 4.9.4. Wild Type Phage from GMO-Bacteria and the Concept of Null Segregants

Legal questions regarding the GMO character of phages may arise if GMO bacteria are used to store and/or amplify wild type phages. So far, one could state that the offspring (organisms) of GMOs are themselves also GMOs in turn, and that derivatives such as insulin or monoclonal antibodies derived from GMOs are not GMOs because these are not organisms. However, wild type phages derived from a GMO bacterium are not merely molecules but are themselves organisms within the meaning of genetic engineering law. If these phages show the wild type genome, a non-GMO would be obtained from a GMO, at least technically. The extent to which this is also a non-GMO phage from a legal point of view will have to be clarified by the scientific discourse. This discussion can build on the arguments for the regulatory handling of so-called “null segregants” (GMO-offspring without the genetic manipulation of the parent generation [117,118,119]), but it must be kept in mind that the previous discussion on null segregants only concerned cases where parent generation and filial generation belong to the same biological species. From a regulatory point of view, this issue would be made less complex if cell-free approaches [69] were used for phage amplification, although the legal significance of phage storage in GMO bacteria would still have to be clarified.

### 4.10. Some Are Advanced Therapy Medicinal Products (ATMP)

The Regulation on Advanced Therapy Medicinal Products (Regulation (EC) No. 1394/2007) covers somatic cell therapeutics, GTMPs, and tissue engineered products. The categorisation of phage therapeutics as ATMP is at most possible for certain phage therapeutics, but not for all phage therapeutics simply because phages are used.

Phage therapeutics are neither somatic cell therapeutics nor tissue engineered products [108]. A requirement for somatic cell therapy medicinal products according to Article 2(1)(a)(2nd point) of Regulation (EC) No. 1394/2007 in conjunction with Annex I Part IV, 2.2 of Directive 2001/83/EC is that the therapeutic product consists of cells or tissues. The same requirement is defined in Article 2(1)(b)(1st sentence) of Regulation (EC) No. 1394/2007 for tissue-engineered products. This does not apply to phages as viruses, and there is no legal equalisation of viruses with cells for ATMP. Rather, it can be seen from the purpose of the ATMP regulation that cell-based ATMP are to be tested for viral safety [120] or that viruses are independently assessed as vectors in the context of gene therapeutics.

Regarding the question of whether phage therapeutics are gene therapeutics according to Article 2(1)(a)(1st point) of Regulation (EC) No. 1394/2007 in conjunction with Annex I, Part IV, 2.1 Directive 2001/83/EC, a differentiated appraisal is necessary. A characterising feature of a gene therapy product is that it contains an active ingredient that, in turn, contains or consists of a recombinant nucleic acid. Phage therapeutics based on wild type phages do not fulfil this requirement. Such phage-based therapeutics are therefore also not gene therapeutics in the sense of pharmaceutical law. Also, wild type phages derived from GMO bacteria are not GTMPs because they do not contain a recombinant nucleic acid (whether these phages are GMOs has to be clarified individually, Section 4.9.4). Also, according to Art. 4(29) of the (extended) definition of GTMP in the EU Commission‘s proposal for the revision of Directive 2001/83/EC, wild type phages would not be GTMPs because they neither edit the host genome in a sequence-specific manner nor contain a recombinant or synthetic nucleic acid.

For genetically modified phages, however, a different legal classification results. As a matter of fact, genetically modified phages contain a mutated nucleic acid and/or a recombinant nucleic acid, and they are used in or administered to humans, as required by the definition of gene therapeutic. As shown above (Section 4.9.2 and Section 4.9.3), mutated phages do not contain recombinant nucleic acid, so genetically modified phages that contain only mutated (and wild type) nucleic acids cannot be GTMP. This legal assessment may change in the future due to the revision of the legal definition of GTMP, which is currently in progress [44].

With respect to the legal classification of genetically modified phages containing recombinant nucleic acids, it should be noted that the legal GTMP definition also requires that the recombinant nucleic acid is used (a) to regulate, repair, replace, add, or remove a nucleic acid sequence and (b) that the therapeutic, prophylactic, or diagnostic effect relates directly to the recombinant nucleic acid sequence it contains, or to the product of genetic expression of this sequence.

At least, the recombinant phage nucleic acid regulates and/or replaces bacterial nucleic acid sequences in the bacteria to be targeted. Since so far in gene therapeutics only cells of humans are manipulated (treated) by the administered recombinant nucleic acid and not the pathogenic bacteria colonising humans, it will have to be discussed with regard to phage therapeutics containing recombinant phages whether the artificially controlled regulation/replacement of a nucleic acid sequence of the pathogenic bacteria present in/on the human body is sufficient as a legal requirement for a GTMP. The discussion will therefore focus on answering the question of whether the genetic manipulation of these pathogenic bacteria on/in humans is to be treated legally the same as the genetic manipulation of genetically pathogenic human cells (cells with human DNA).

To answer this question, it may be helpful to refer again to the CJEU judgment in Case C-308/11 [83]. In this case, the CJEU elaborated that the direct effect of the therapeutic agent in question may not be on human cells, but on any cells present in/on the human body, such as bacteria. If this assessment is applied to phage therapeutics, then these would be GTMPs within the meaning of Regulation (EC) No. 1394/2007 (same conclusion, but without the dogmatic reasoning discussed here: [40,108]), provided that, as one can assume on the basis of the healing processes, physiological functions are also restored, corrected, or influenced. This classification of recombinant phages as GTMP explained here is also in line with the so far only classification of a genetically (by CRISPR) modified phage as GTMP by the EMA [121]. However, an EMA classification has no prejudicial effect on future classifications.

### 4.11. Some Are Biological Medicinal Products (Biologicals), Some Could Be, Some Are Not

Pharmaceutical legislation has specific provisions for biological medicinal products (biologicals), cf. Annex I, Part 1, 3.2.1.1, b) sentences 3–5 of Directive 2001/83/EC.

Phage therapeutics have already been addressed as biologicals [3,80,108,122], although no reference has been made to the wording of the law, nor has a dogmatic reasoning been given. At best, reference is made to the conference on phage therapeutics at EMA in 2015, during which this statement was made in a presentation [123]. However, phage therapeutics—even if this may seem obvious at first glance—are not biological medicinal products per se in the sense of pharmaceutical law [100].

The definition of biological medicinal product in Annex I, Part 1, 3.2.1.1, b) sentence 5 of Directive 2001/83/EC conclusively lists biological medicinal products for human use. Accordingly, biological medicinal products include: “*immunological medicinal products and medicinal products derived from human blood and human plasma […]; medicinal products falling within the scope of Part A of the Annex to Regulation (EEC) No. 2309/93; advanced therapy medicinal products […].*”. As shown, phage therapeutics are neither immunological medicinal products nor blood-derived medicinal products. Medicinal products covered by Part A of the Annex to Regulation (EEC) No. 2309/93 (*NB! Regulation (EEC) No. 2309/93 is no longer in force since 19.11.2005. It has been replaced by Regulation (EC) No. 726/2004. According to Article 88 (sentence 2) of Regulation (EC) No. 726/2004 references to the repealed regulation are deemed to be references to Regulation (EC) No. 726/2004. The medicinal products referred to in the repealed Annex, Part A of Regulation (EEC) 2309/93 can be found with identical wording in: Annex I, No. 1 Regulation (EC) No. 726/2004*) include medicinal products prepared by techniques of recombinant DNA, controlled expression in prokaryotes and eukaryotes, including transformed mammalian cells, genes encoding biologically active proteins, and hybridoma- and monoclonal antibody-based techniques. This enumeration of techniques does not apply to wild type phages (at least to those amplified in wild type bacteria). At most, the enumeration may be relevant for recombinant phages that would also be ATMP for bacterial combating purposes. Thus, phage therapeutics based on recombinant phages can be biological medicinal products under Annex I, Part 1, 3.2.1.1, (b)(sentence 5) of Directive 2001/83/EC.

A different view for wild type phages may result when wild type phages are stored and/or amplified in GMO bacteria (Section 4.9.4). In this case, it is necessary to examine the extent to which these wild type phages are medicinal products manufactured with technologies of recombinant DNA within the meaning of Annex I No. 1 of Regulation (EC) No. 726/2004. Although the phages as such are not genetically modified, their manufacture (amplification) involves the technology of recombinant DNA for the required GMO bacteria. If one considers that the jurisdiction of the CJEU allowed an indirect pharmacological effect to be sufficient for the determination of the pharmacological action (Section 2.3), then it will have to be clarified whether an indirect use of the technology of recombinant DNA is also to be legally regarded as a direct use if this indirect use is an inherent part of the manufacture. If this is the case, then the consequence would be that wild type phages would also be biologicals.

It should be taken into account that the exhaustive list of biological medicinal products concerns a different aspect than the question of whether a substance is a suitable starting material for a biological medicinal product (cf. Annex I, Part 1, 3.2.1.1, b) sentence 2 of Directive 2001/83/EC). The legislator obviously assumes that a biological starting material does not automatically lead to a biological medicinal product. If this link existed, the legal wording would have to state that a biological medicinal product always results from a corresponding substance. Phages are also not explicitly mentioned as a suitable starting material. However, the list of suitable starting substances is not exhaustive (“such as”). Therefore, phages can be a starting material for a biological medicinal product because phages match the substances already explicitly mentioned there in terms of origin and material properties. However, as phage-based medicinal products are not mentioned in the exhaustive list of biological medicinal products, the use of phages as a starting material does not automatically lead to a biological medicinal product. According to Article 4 (14) of the Commission’s proposal, in the future, biologicals would no longer be defined by an exhaustive list but, among other things, by an active substance that is produced by or extracted from a biological source [44]. Provided that medicinal phages are isolated from bacteria, this requirement would be fulfilled. However, it is legally controversial that Annex I of the Commission’s proposal continues to include the same exhaustive list of biological medicinal products that is included in the current Directive 2001/83/EC. If the future Annex I is applied to answer the question of whether a phage-based medicinal product is a biological medicinal product, then it can also be argued that phage-based therapeutics would not be biological medicinal products in the legal sense. If, from the perspective of medical research and technical manufacturing, the specific regulations for the manufacture and testing of biological medicinal products should apply unambiguously to phage-based medicinal products in the future, then phage-based medicinal products should be explicitly included in the list of biological medicinal products in the (future) Annex I.

### 4.12. Conclusion on the Classification: Not Specifically Regulated Medicinal Product with One Exception

Today, therapeutics for the treatment of bacterial infections based on wild type phages (yielded from non-GMO bacteria) cannot be assigned to any specifically regulated class of medicinal products under EU law. They are therefore “simple” medicinal products (by function) within the meaning of Article 1(2)(b) Directive 2001/83/EC (cf. Table 1).

Only medicinal products based on recombinant phages (and maybe wild type phages yielded from GMO bacteria) can fall into the specifically regulated medicinal product class of ATMP, in the form of GTMP, Article 2(1)(a)(1st point) of Regulation (EC) No. 1394/2007 in conjunction with Annex I, Part IV, 2.1 of Directive 2001/83/EC. Thus, medicinal products based on such GM-phages also belong to biological medicinal products according to Annex I, Part 1, 3.2.1.1, b) sentences 3–5 of Directive 2001/83/EC.

## 5. Significance of the Medicinal Product Type, the Preparation and Application Scenarios for the Legal Handling of Phage Therapeutics

### 5.1. No Prohibition of Phage Therapy

At the level of EU law, there is no prohibition of phage therapy. Besides, the EU has no legislative competence to prohibit such a medicinal product. This competence lies within the Member States. However, in view of the successes achieved to date and the lack of evidence of a harmful effect of phages when used as intended, such a prohibition at the Member State level seems unreasonable. In addition, phages are not derived from a controversially discussed origin, as, e.g., is the case with human embryonic stem cells, so that no restrictions on phage therapy can also be identified at the Member State level regarding the source of the phages.

### 5.2. Amplification of Phages in Phage Banks or a Specialised Facility without Preparation of the Medicinal Product in Final Dosage Form

The amplification of phages in therapeutically required quantities by a phage bank or a specialised facility without the manufacture of the ready-to-use phage medicinal product is the manufacture of active substances (Section 4.2).

For sales, generally for dispensing, of these phages to manufacture ready-to-use phage-based medicinal products, no stand-alone marketing authorisation is required under EU law. Therefore, as long as they are not ready-made medicinal products, placing phages on the market does not require (pre-)clinical studies. However, the operation of the phage bank or a respective specialised facility that amplifies phages for medicinal use must be done according to the GMP standard, Article 46b(1) of Directive 2001/83/EC. For the operation of this kind of phage bank, the specifications from “ICH Topic Q 5 D” [124] may also be relevant. It provides specifications for the derivation and characterisation of cell substrates used for the production of biotechnological/biological products. At the level of EU law, there are no legal exceptions that exempt phage banks or respective specialised facilities from these GMP requirements.

Only the amplification of phages within a pharmacy as part of the patient-specific preparation of a phage-based medicinal product (i.e., as a magistral formula) is not covered by this GMP requirement under EU law, because Directive 2001/83/EC does not apply to patient-specific medicinal products prepared in pharmacies in accordance with Article 3(1) of Directive 2001/83/EC. In this case, it is necessary to check in each EU Member State whether there are national GMP requirements for this setting. The GMP requirement also does not apply to phage banks or other facilities that store or produce phages for scientific purposes without their use in medicinal products (cf. Table 2).

### 5.3. Centralised, Patient-Independent, Industrial, Uniform Preparation in Advance

If uniform phage therapeutics are manufactured in advance outside pharmacies industrially or by using an industrial method, Directive 2001/83/EC and its implementing acts in the EU Member States are applicable in accordance with Article 2(1) of Directive 2001/83/EC (cf. Table 2).

According to Article 40 of Directive 2001/83/EC, a manufacturing authorisation is required for the manufacture of wild type phages and ATMP-phage therapeutics, which is issued by the authorities in the EU Member State. For this, according to Article 46(f) of Directive 2001/83/EC, the GMP standard is required. As a consequence, for the manufacture of active pharmaceutical ingredients and medicinal products, compliance with the quality management system according to GMP is in principle—with the exceptions provided in the law (see below)—legally required (cf. Article 46(f); 46b(1) Directive 2001/83/EC). The requirements of the GMP standard are described in the EU GMP Guideline published by the European Commission, Article 47 Directive 2001/83/EC. However, the current GMP standards do not have specific requirements for the manufacture of phage therapeutics. The extent to which the current GMP standard adequately describes the production of phage therapeutics in terms of opportunities and risks must therefore primarily be discussed in technical and medical context. If a need for adaptation is identified, regulatory adjustments are possible, for which the “GMP/GDP Inspectors Working Group” (GMDP IWG) based at the EMA must become active. In Art. 47 of Directive 2001/83/EC, EU law also expressly stipulates that the GMP specifications are to be revised if this becomes necessary due to technical and scientific progress.

With regard to recombinant phage-based ATMP, it is important to bear in mind that these are ATMP and that specific GMP requirements have been established in accordance with Article 5 of Regulation (EC) No. 1394/2007 for ATMP [125]. But once again, it is a separate technical question, and therefore not investigated further here, whether these existing GMP specifications for ATMP also adequately reflect the requirements for phage medicine.

If a manufacturer manufactures phage-based medicinal products, but does not keep the required phages in stock in their own phage bank and does not amplify them, but obtains the phages from a third-party supplier, then the manufacturer of the ready-to-use phage medicinal product is obliged under EU law to use only active substances that have been manufactured in accordance with GMP, Article 46(f) of Directive 2001/83/EC.

The ISO 9001 standard sometimes cited for the preparation of phage therapeutics, by contrast, is not legally binding. GMP and ISO both describe quality management systems. The ISO 9001 standard aims to guarantee the quality of products in general. In contrast to the GMP standard, however, the ISO standard is not specifically tailored to the pharmaceutical sector but covers products in general and thus active ingredients and medicinal products. In addition, unlike the GMP standard, the ISO standard is not legally required in the pharmaceutical sector. Therefore, the ISO standard does not play a role in the legally compliant translation of phage therapy.

The question of whether Regulation (EC) No. 726/2004 or the respective national pharmaceutical legislation is relevant for the marketing authorisation must be answered on the basis of Article 3(1) in connection with Annex I or Article 3(2) of Regulation (EC) No. 726/2004. Phage therapeutics based on wild type phages do not fulfil any of the types of medicinal products, manufacturing methods, and/or indications listed in Annex I. Thus, such phage therapeutics are not subject to the mandatory authorisation under Article 3(1) of Regulation (EC) No. 726/2004. However, insofar as wild type phages are amplified in GMO bacteria, it is necessary to examine whether a mandatory central authorisation is required for these phages (Section 4.9.4).

If a phage therapy medicinal product based on wild type phage is nevertheless to be centrally authorised, an optional authorization in accordance with Article 3(2) of Regulation (EC) No. 726/2004 may be considered. For this purpose, the applicant for the specific phage therapy medicinal product must demonstrate that it contains an active substance that was not approved in the EU on 20 May 2004, or that it represents a significant innovation in therapeutic, scientific, or technical terms, or that the granting of an approval under this Regulation at the EU level is in the interest of patient health.

If neither mandatory nor optional EU authorisation is relevant, the wild type therapeutic must be authorised under the pharmaceutical legislation of the EU Member State in which this therapeutic is to be placed on the market.

In contrast, pre-made phage ATMP based on recombinant phages are subject to mandatory authorisation according to Article 3(1) in conjunction with Annex I of Regulation (EC) No. 726/2004, since these are both ATMP and medicinal products for whose preparation recombinant DNA techniques are used.

Irrespective of the responsible authority for granting the marketing authorisation, the granting is linked to the successful completion of preclinical and clinical studies to demonstrate efficacy and safety (e.g., Article 6 et seqq. of Directive 2001/83/EC, Article 3(1), (2) of Regulation (EC) No. 726/2004).

With regard to the legal framework for the patient-specific preparation of phage-based therapeutics that are legally ATMP due to the genetic modification, a distinction must be made as to whether this takes place in a pharmacy (see Section 5.4.3) or by pharmaceutical companies outside of pharmacies (see Section 5.6.2).

### 5.4. Decentralised Patient-Specific Preparation on Demand in (Hospital Based) Pharmacies

#### 5.4.1. Legal Framework

Since, for the technical reasons shown, it is not to be expected that phage therapeutics will be prepared in a community pharmacy, the patient-specific preparation will take place in the interaction of hospitals (diagnosis, prescription, medical care) and the associated hospital pharmacies (preparation of the medicinal product according to the prescription). As EU pharmaceutical law does not distinguish between community pharmacies and hospital pharmacies, both types of pharmacies are also subject to the same legal framework from the perspective of EU law, provided they are legal pharmacies under the laws of the Member States.

The regulatory framework to be followed regarding the preparation of phage-based medicinal products within the hospital pharmacy is determined by whether the therapeutic is based on wild type phages or ATMP-phages.

#### 5.4.2. Wild Type Phages

If patient-specific medicinal products with wild type phages are prepared in a hospital pharmacy according to a medical prescription, then, according to Article 3(1) of Directive 2001/83/EC, Directive 2001/83/EC is not applicable (so-called *magistral formula*). This applies to both the setting in which the phages are stored and amplified in a phage bank in the hospital pharmacy itself and to the setting in which the required phages (as an active substance) are obtained by the hospital pharmacy from an external phage bank offering this service or by a facility specialised in the amplification of phages. In both cases, the ready-to-use medicinal product is prepared in the pharmacy, and this is the focus of Article 3(1) of Directive 2001/83/EC. The legal consequence of this derogation of Directive 2001/83/EC is that these phage-containing medicinal products are not regulated at the level of EU law, i.e., there is no obligation for a manufacturing authorisation, nor obligation to prepare under GMP, nor authorisation for placing on the market under EU law. The legal requirements for the preparation are based only on the law of the respective Member State in which the preparation takes place (cf. Table 2).

Under EU law, there is no option for marketing authorisation at the EU level. Directive 2001/83/EC is not applicable, Article 3(1) and 6(1). Therefore, these therapeutics are also not covered by Regulation (EC) No. 726/2004, since Regulation (EC) No. 726/2004, according to its Article 2 and Article 3, only applies to medicinal products covered by Directive 2001/83/EC. Therefore, under EU law, there is also no obligation for phage-based medicinal products prepared in pharmacies to undergo (successful) (pre-)clinical testing prior to their regular application. This obligation exists only for medicinal products that are to be authorised. Another question is to what extent these kinds of studies may be carried out “voluntarily“, i.e., without them being usable for a marketing authorisation application. The extent to which marketing authorisation and trials are required must therefore be clarified in each individual Member State.

Finally, patient-specific medicinal products prepared in pharmacies based on wild type phages cannot be regulated uniformly by the EU, and each Member State can enact its own regulations. The EU legislator has established a harmonised EU regulation for individualised medicinal products only for ATMP within the framework of the hospital exemption. However, this provision is not applicable to therapeutics based on wild type phages but to therapeutics based on ATMP-phages, which are ATMP (Section 4.10 and Section 5.6.2).

#### 5.4.3. Magistral ATMP-Phages outside the ATMP-Regulation?

If phage pharmaceuticals that fulfil the legal definition of GTMP are prepared, this technically qualifies as the preparation of ATMP (Section 4.10). This also applies—again, only technically speaking—to the preparation of such GTMP in a (hospital) pharmacy. However, this technical view does not correlate with the legal appraisal.

In principle, the preparation of patient-specific ATMP is regulated by the so-called hospital exemption according to Article 28 of Regulation 1394/2007, Article 3(7) of Directive 2001/83/EC). ATMP that are legally covered by the hospital exemption are not authorised by the EU—unlike patient-independent, ready-made ATMP—but by the Member States, albeit to the standard of EU-authorised ATMP (Section 5.6.2). It is questionable whether these legal requirements of EU law also apply to patient-specific ATMP that are prepared as magistral formulas in a pharmacy. This is due to the fact that Article 3(1) of Directive 2001/83/EC explicitly excludes all (!) magistral formula medicinal products from the scope of Directive 2001/83/EC. This general exception indicates that the magistral formula provision also covers ATMP [126]. Therefore, it is not relevant that Article 3(7) of Directive 2001/83/EC lays down rules for patient-specific ATMP, whereas these EU requirements for patient-specific ATMP are to be established by the Member States. If the EU legislator wants magistral formula ATMP to be covered by the EU harmonised hospital exemption in the same way as patient-specific ATMP that are prepared outside pharmacies by pharmaceutical companies, then the EU legislator must first limit the magistral formula exemption itself, i.e., explicitly exclude patient-specific ATMP from the magistral formula. At the moment, however, one must assume that patient-specific ATMP in general [126] and therefore also phage-based ATMP prepared in pharmacies in particular are not covered by EU law.

Even if the magistral formula medicinal products are technically placed on the market, no marketing authorisation is required under EU law, because, according to Art. 6(1) of Directive 2001/83/EC, only medicinal products that fall within the scope of Directive 2001/83/EC require a marketing authorisation under EU law. However, as shown, magistral formula medicinal products are excluded from the scope of the Directive. The handling of these medicinal products, even if they are technically ATMP (but not regulated as such), is therefore governed by the pharmaceutical legislation of the EU Member States. From the perspective of EU law, the patient-specific preparation of (phage-based) ATMP in pharmacies is therefore different from the patient-specific preparation of (phage-based) ATMP by pharmaceutical companies other than pharmacies (Section 5.6.2).

### 5.5. Patient-Specific Preparation by Physicians and Application in Medical Practices

Depending on the technical requirements, physicians can also prepare medicinal products within their doctors’ office. If physicians use these in-house prepared medicinal products on their patients, these medicinal products are not considered to be placed on the market from an EU legal perspective. This in turn means that the preparation and administration of these medicinal products are not covered by EU law, because Directive 2001/83/EC and Regulation (EC) No. 726/2004 only cover medicinal products that are placed on the market. The legal framework for medicinal products prepared by physicians in this way is therefore determined by the law of the respective EU Member State. For example, in Germany, physicians are allowed to prepare medicinal products for administration to their patients. The extent to which a manufacturing authorisation is necessary for physicians in this constellation depends on the type of medicinal product. The situation is different in Austria, for example, where physicians are not allowed to prepare medicinal products, not even patient-specific medicinal products, for their patients. For the Member States in which the preparation of medicinal products by physicians is permissible, the following aspects apply to phage-based medicinal products:

If phage therapeutics, due to technical progress, are prepared and administered (eventually using phages from third parties) by a physician in a medical practice on patient-specific cases, Directive 2001/83/EC does not apply; neither to therapeutics based on wild type phages nor to those based on ATMP-phages. This derogation of Directive 2001/83/EC is not a case of “named patient use” according to Article 5(1) Directive 2001/83/EC, but rather these cases are outside the scope of the Directive according to Article 2 of Directive 2001/83/EC. Directive 2001/83/EC is not applicable because the (phage) medicinal products prepared and applied in this way by physicians are not placed on the market in the legal sense. However, according to Article 2(1) and 6(1) of Directive 2001/83/EC, this condition is a prerequisite for the applicability of Directive 2001/83/EC.

The details of the term “placing on the market” can only be described briefly here. Placing on the market within the meaning of pharmaceutical law typically requires a change in the power of disposal over the medicinal product. This is the case, for example, when the manufacturer supplies the medicinal product to the wholesaler or pharmacist. Subsequently, the wholesaler or pharmacist can in turn dispense the medicinal product to other people. If a physician applies a (phage) medicinal product prepared by him/her to a patient, then the patient cannot dispense the phage medicinal product to another person following the treatment, so there is no placing on the market.

Complex legal issues arise when physicians prepare therapeutics that consist of a medicinal product component (phage) and a medical device component. This is because Regulation (EU) No. 2017/745 for medical devices defines placing on the market as making available for the market (for the first time). Regulation (EU) No. 2017/745 defines this as any supply of a device for distribution, consumption, or use (Article 2(27) and (28). This definition is broader than the understanding of placing on the market according to Directive 2001/83/EC, so that possibly the application of a medical device manufactured by physicians can already fall within the scope of Regulation (EU) No. 2017/745, possibly as a custom-made device according to Article 2(3). Since placing on the market does not have the same meaning in Directive 2001/83/EC and Regulation (EU) No. 2017/745, it is necessary to clarify for the complete product whether at least the medical device component is regulated under EU law.

If, at least for the phage medicinal products prepared and administered by physicians, Directive 2001/83/EC does not apply, then there is no obligation under EU law to link this preparation to an authorisation and no obligation to comply with the GMP standard. EU law also does not impose an obligation to obtain a marketing authorisation, because Regulation (EC) No. 726/2004 requires that the product in question be a medicinal product covered by Directive 2001/83/EC. In addition, Regulation (EC) No. 726/2004 is based on placing on the market in the sense of pharmaceutical law, a condition that is not fulfilled by (phage) medicinal products prepared and administered by physicians. Since there is no legal obligation to grant marketing authorisation, there is no legal obligation to (pre-) clinically test these phage-based medicinal products prior to their use (cf. phage medicinal products manufactured in pharmacies, Section 5.4).

Since Directive 2001/83/EC does not apply to medicinal products prepared and administered by physicians, even the preparation and administration of phage medicinal products containing ATMP-phages are not regulated by Directive 2001/83/EC. The hospital exemption, which should be applicable to ATMP-phages as ATMPs, does not apply either, because the hospital exemption is only effective via Article 3(7) of Directive 2001/83/EC; however, Directive 2001/83/EC is, as described, not applicable. A further question to be clarified on a case-by-case basis is whether Member State regulations apply to these phages containing ATMP, particularly, to what extent genetic engineering law of the EU and the Member States has to be considered (cf. Table 2).

Even if, under EU law, there is no obligation to obtain a manufacturing authorisation or a marketing authorisation for phage therapeutics prepared individually for patients by physicians, this does not correspond to a prohibition of this form of preparation and application at the level of EU law. Physicians can prepare these medicinal products and administer them to their patients, provided that they comply with any regulations that may exist in the Member States; EU law, however, does not stipulate any requirements in this regard.

### 5.6. (De)Centralised Patient-Specific, Industrial Preparation on Demand, outside Pharmacies

#### 5.6.1. Legal Framework and Medicinal Products Based on Wild Type Phages

From a technical point of view, patient-specific phage therapeutics can also be prepared by pharmaceutical companies. In this constellation, Directive 2001/83/EC, respectively, the Member States’ implementations are applicable. It is not a preparation in a pharmacy, so the exemption provision of Article 3(1) of Directive 2001/83/EC is not relevant. Instead, within the meaning of Article 2(1) of Directive 2001/83/EC, it is a medicinal product that is either manufactured industrially or involves an industrial process.

Even if the individualised therapeutic products are each made on their own, this is an industrial manufacture or a manufacture with an industrial process within the meaning of Article 2(1) of Directive 2001/83/EC. The CJEU has emphasised that this requirement is to be interpreted broadly. Accordingly, an industrial process is generally characterised by a succession of operations, which may be in particular mechanical or chemical, in order to obtain a significant quantity of a standardised product [127,128]. This also applies to a pharmaceutical company preparing individual phage medicinal products, since the pharmaceutical company also (eventually by means of a magisterial instruction and/or in compliance with the specifications in the pharmacopoeia, Section 5.7 and Section 5.8) prepares individual (phage) medicinal products from the required components in a standardised manner by means of a previously defined succession of preparation steps (operations), whereby only the phages required in each case and the dosage are varied in relation to the patient. With this definition of the term “industrial”, the preparation in a pharmacy can also be industrial. However, this is irrelevant because the preparation of (phage) medicinal products in pharmacies is excluded from the scope of application of Directive 2001/83/EC, irrespective of whether this is an industrial manufacture (cf. Table 2).

Because Directive 2001/83/EC applies to the customised preparation of phage-based medicinal products by pharmaceutical companies, the same regulations regarding manufacture and placing on the market apply as in the case of industrial, patient-independent preparation in advance (Section 5.3).

In this setting, however, technical, and thus legal difficulties may arise regarding the marketing authorisation. For an authorisation, among other things, successful clinical studies are necessary. This is a requirement that typically applies to a medicinal product that is manufactured in the same way each time. However, if a medicinal product is prepared on a patient-specific basis and also only on demand, the question arises: What should be clinically tested in advance for this medicinal product? For this setting, neither Directive 2001/83/EC nor Regulation (EC) No. 726/2004 provide for an exemption from the obligation to obtain marketing authorisation, and thus there are no exemptions from the requirement of successful (pre-) clinical trials.

To address this issue, for patient-specific phage medicinal products, the so far not permitted regulatory concept of the “biological master file” could possibly be useful, whereby marketing authorisations are granted for individual phages or homologous groups of phages. From such (authorised) phages, the individually required medicinal product could be manufactured using further excipients without the need for further independent authorisation [94].

In addition, it can be assessed whether the regulation of the preparation of individual phage therapeutics by replacement of phages (as an active ingredient) in an already authorised phage medicinal product can be based on the regulatory concept for changes to the authorisation (extension/variation) within the meaning of Regulation (EC) No. 1234/2008, which may need to be amended accordingly [108,122].

#### 5.6.2. Phage-Based ATMP

If pharmaceutical companies (not pharmacies) prepare and market patient-specific phage-based medicinal products that legally constitute ATMP, then—in contrast to the preparation in a pharmacy (see Section 5.4.3)—the hospital exemption of the ATMP Regulation is applicable. The hospital exemption for patient-specific ATMP was established by the EU legislator for patient-specific ATMP within the framework of Regulation (EC) No. 1397/2004. In short, for certain, i.e., patient-specific ATMP (as opposed to patient-independent, pre-manufactured ATMP), the hospital exemption constitutes an exemption to the mandatory authorisation of ATMP by the EU for the economic area of the EU. The hospital exemption allows patient-specific ATMP to be manufactured only in a specific Member State and to be placed on the market only there, for which an authorisation from the respective Member State authority is required, but no authorisation by the EU. For this, Article 28(2) of Regulation (EC) No. 1394/2007 introduced Article 3(7) of Directive 2001/83/EC, according to which the manufacture of patient-specific ATMP is subject to authorisation by the competent authority of the Member State. The Member States shall ensure that the national traceability and pharmacovigilance requirements, as well as the quality standards, are equivalent to those applicable at the EU level for ATMP for which a marketing authorisation is required in accordance with Regulation (EC) No 726/2004. As a result of this legal equalisation of the quality standards, the manufacture of patient-specific ATMP based on recombinant phages must be approved, on the one hand, by the authority of the Member State in which the manufacture takes place. On the other hand, the preparation must comply with the GMP standard, because the industrial pre-manufacture of ATMP approved by the EU must also meet the GMP standard (Section 5.3). Due to the legal alignment for traceability and pharmacovigilance, an environmental risk assessment according to Annex I, Part 1, 1.6 of Directive 2001/83/EC is also required if the ATMP-phage is a GMO, which must be decided based on genetic engineering law (Section 4.9.1). The extent to which further preclinical and clinical studies are necessary prior to regular preparation and application within the scope of the hospital exemption must be addressed in the Member State in which the hospital exemption is to be used. As the authorisation under the hospital exemption is granted by a Member State for its territory, the ATMP concerned (with recombinant phages) can only be placed on the market in that Member State.

### 5.7. The Meaning of the Discussion on the Magistral Preparation

To provide a regulatory framework for the therapeutic use of patient-specific phage therapeutics, the concept of magistral preparation is discussed, at least to ensure the quality of the preparation [31,33,42,129,130,131]. This discussion does not concern all cases of the “*magistral formula*” covered by Article 3(1) of Directive 2001/83/EC, but only a part of it, since every magistral preparation is a *magistral formula*, but not every *magistral formula* is a magistral preparation. Both terms are—at least in some EU Member States—not 100% identical.

Magistral formulation generally means that a medicinal product is individually prepared for a patient in a pharmacy in accordance with a physician’s prescription with the composition specified by the physician in the prescription. The term is used as a generic term for individual preparations and magistral preparations.

An individual preparation is based on an individual prescription, which in turn is based on the experiences of the prescribing physician. Thus, an individual prescription is typically not based on compounds that have already been generally reviewed pharmaceutically for the plausibility of their pharmaceutical formulation (e.g., stability, dosing, application method). Therefore, within the risk assessment for the preparation of this subtype of the magistral formula, the pharmacist has the duty to scrutinise the prescription for the plausibility of its pharmaceutical formulation in detail every time it is prepared [132].

The magistral preparation is also prescribed by a physician. However, in contrast to the individual preparation, the magisterial preparation is based on standardised and published compositions that have been reviewed by the pharmaceutical sciences. Except for patient-specific aspects such as the dosage, the magisterial preparation has therefore already been checked for the plausibility of its pharmaceutical formulation.

The effect of the magistral preparation is that, on the one hand, a consistent product quality can be ensured despite the individual character of the medicinal product. On the other hand, the magistral preparation reduces the workload for the pharmacist, since the effort for plausibility testing is reduced in comparison to individual formulations due to the consensual, scientifically proven origin of the magistral formulation. However, attention is needed when it comes to the discussion about the magistral formulation and its meaning for the efficacy and safety of phage-based medicinal products.

The magistral preparation, or the establishment of the magistral formulation for certain phage-based medicinal products, does not require preclinical or clinical studies to prove efficacy and safety as in the context of clinical studies. Instead, magistral preparation concerns aspects of manufacturing quality. Thus, the magistral preparation has no relevance for the regulatory classification of phage-based medicinal products or for questions of whether the GMP standard must be applied for the preparation from a legal point of view.

### 5.8. The Meaning of the Incorporation of Phages into the European Pharmacopoeia

In June 2021, the European Pharmacopoeia Commission adopted a resolution to establish a general chapter on phage therapy active substances for human and veterinary medicinal products. For this purpose, the “Working Group Bacteriophages “was commissioned with the preparation of this draft, which was released for public consultation at the “European Directorate for the Quality of Medicines & HealthCare” of the Council of Europe until the end of June 2023 [42,133]. However, phage therapeutics will not be (pre-) clinically tested by inclusion in the European Pharmacopoeia, but rather quality standards for active substances or dosage forms will be standardised.

The incorporation of phages into the European Pharmacopoeia has no significance for the legal appraisal of the type of medicinal product, the obligation to obtain a marketing authorisation or the requirement for a manufacturing authorisation, since the relevant regulations on classification and authorisation are not based on the incorporation of an active ingredient into the pharmacopoeia. A different question is to what extent pharmacists and other pharmaceutical manufacturers are legally obliged to comply with requirements of the European Pharmacopoeia when manufacturing a phage-based medicinal product, provided that phages are included in the pharmacopoeia. For this, numerous obligations can be found in Directive 2001/83/EC (e.g., Annex I, Introduction and General Principles, (5); Annex I, Part I, 3.2., (5); Annex I, Part I, 3.2.1.1., a). However, phage therapeutics can also be manufactured without the pharmacopoeia being amended or the magistral formulation being available. There is no prohibition at the level of EU law that links manufacturing to such conditions. Directive 2001/83/EC only requires that the specifications of the pharmacopoeia are met if these standards are available; the directive does not stipulate that a medicinal product can only be manufactured if there are (already) accepted pharmaceutical standards for that medicinal product. This is also in line with Council of Europe Resolution CM/Res(2016)1 (cf. Chap. 8) [132].

### 5.9. Phages in Compassionate Use, Named Patient Use, Off-Label Use & Experimental Therapy

Compassionate use (according to the EU legal meaning in Article 83 of Regulation (EC) No. 726/2004, i.e., the possibility of using medicinal products that are either the subject of an application for a marketing authorisation or are undergoing clinical trials), off-label use, and named patient use (Article 5(1) of Directive 2001/83/EC) are considered as a basis for phage therapy [3,95,100,130,134]. These concepts reflect the view of Article 37 of the Helsinki Declaration, according to which, under the condition that no (standard) therapy is available, unproven medical interventions should also be permissible if this unproven treatment offers hope of saving life, re-establishing health, or alleviating suffering. Therefore, these concepts can only be utilised in the exceptional cases specified by law. If the perspective for phage therapy is the regular application in medical care, then these concepts cannot provide a basis for phage therapy.

Finally, the concept of experimental therapy (right-to-try) can be considered for phage therapy. In a strict sense, this is defined as the use of treatments that are not medical standard in patients who have received the available conventional therapy without therapeutic success, whereby it is not a question of off-label use or cases of Article 5(1) of Directive 2001/83/EC. This includes the use of experimental substances that are legally not (yet) considered medicinal products. This, in turn, can be individual, experimentally manufactured, and administered phages, for which the necessary dosage form (which is the medicinal product in the legal sense, Section 4.2) is still to be determined. These cases of experimental therapy are not regulated by EU law; in particular, they are not cases of Article 5(1) of Directive 2001/83/EC, because it is not a question of an exemption from the application of Directive 2001/83/EC. The legal framework is therefore regulated by the Member States. However, this experimental treatment option only concerns individual patients and is therefore not suitable for the standard application of phage therapy.

Although the preparation and application of phage medicinal products within the scopes of Article 3(1) of Directive 2001/83/EC, the hospital exemption of Regulation (EC) No. 1394/2007, and of physician-made medicinal products are also regulatory exceptions compared to the standard case of the application of medicinal products with marketing authorisation and manufacturing authorisation. However, these exceptions, unlike compassionate use, off-label use, or named patient use, are not limited to patients who have been offered all available therapies without success according to conventional medicine. Phage therapeutics that are prepared or used in accordance with Article 3(1) of Directive 2001/83/EC, with the hospital exemption, or in accordance with the rules for physician-made medical products are not limited to exceptional medical cases, emergencies, cases without an available therapeutic agent, etc., but can be the first medicinal product applied.

## 6. Phage Therapy and Nagoya Justice

As soon as phages can be commercialised on a large scale, there will be a discussion about who owns phages or phage genomes, especially with respect to phages collected in the global south, but whose economic exploitation is intended to take place in the industrialised nations of the global north. This discussion should clarify the extent to which patenting phage therapy related inventions is morally justifiable when the therapeutic benefits are not geographically available to all affected individuals in the same way. In this context of ownership, patentability, and economic exploitation, the Nagoya Protocol, an international treaty governing access and benefit-sharing for the use of biodiversity and genetic resources, may be relevant [135,136].

With respect to phages, it will first be necessary to clarify the extent to which the UN Convention on Biological Diversity (CBD) covers phages, since the Nagoya Protocol only applies to resources covered by the CBD. The CBD, in turn, does not explicitly refer to viruses as a specific resource. However, Article 1 CBD covers genetic resources, which also include genetic material of plant, animal, and microbial origins as well as genetic material of other origin (Article 2). Therefore, it is arguable that viruses and their genetic resources fall within the scope of the CBD and, thereby, also within the scope of the Nagoya Protocol.

## 7. Veterinary Medicine

The technical as well as the legal discussion on the use of phages in veterinary medicine is more advanced compared to human medicine. The EU Regulation on Veterinary Medicinal Products, Regulation (EU) No. 2019/6, explicitly paved the way for the medicinal use of phage therapies for animals. Article 4(43)(a) of Regulation (EU) No. 2019/6 defines a veterinary medicinal product for advanced therapies as, inter alia, a veterinary medicinal product that has been specifically developed for phage therapy. In veterinary medicinal products, phage therapeutics are therefore Veterinary-ATMP, whereas this general classification does not (yet) apply to medicinal products for human use.

Regulation (EU) No. 2019/6 also establishes requirements for the applications for marketing authorisation for veterinary phage medicinal products under Article 8(1)(b) in conjunction with Annex II, V. 1.5.4. A marketing authorisation application needs to include the technical documentation necessary to demonstrate the quality, safety, and efficacy of the veterinary medicinal product. Regarding phage therapeutics, Regulation (EU) No. 2019/6 also specifies the phage banks required or how GM-phages are to be handled. To specify the regulatory requirements for phage therapeutics under Regulation (EU) No. 2019/6, the EMA published the “Guideline on quality, safety and efficacy of veterinary medicinal products specifically designed for phage therapy” in October 2023 [137].

It remains to be seen to what extent the discussion and regulation in veterinary medicines can be transferred to answer the unanswered questions in human medicines.

## 8. Food Sector

Phages can also be used to prevent bacterial spoilage of food, e.g., by EHEC pathogens, listeria, or salmonella. In the EU, the use of phages in the food sector is discussed within food legislation as a separate technical and legal debate [138,139]. However, the use of phages for food preservation is not a matter of pharmaceutical law.

## 9. Regulatory Outlook

The EU Commission‘s proposal to reform Directive 2001/83/EC [44] has not adequately acknowledged the significance of phage therapeutics by allowing specific regulations to be established according to Article 28. The proposed Article 28 is not convincing, because the majority of individualised phage therapies, the focus of phage therapy, would not be covered by this regulatory tool.

Although Article 28 in conjunction with Annex VII of the Commission‘s proposal provides that an adapted framework due to the characteristics or methods inherent to the medicinal product shall be established for phage-containing medicinal products, but only in cases where the medicinal product has a variable composition depending on the specific clinical context [44].

Therefore, the provisions based on Article 28 would not apply to industrially pre-manufactured, i.e., patient-independent, phage therapeutics (wild type and GM) because they do not have a variable composition. Instead, these phage medicinal products would still have to be authorised by either the EU or the Member States, like other medicinal products produced industrially in advance. If EU authorisation is possible, individual technical aspects of quality, safety, and efficacy could be covered by an appropriately revised “Guideline on the evaluation of medicinal products indicated for treatment of bacterial infections” [7] issued by the EMA. In addition, the results of the EMA’s consultation process for the preparation of the “concept paper on the development and manufacture of phage medicinal products for human use” [45] could be relevant in the future.

Despite their variable composition, the new provisions under Article 28 would also not apply to phage therapeutics manufactured individually as magistral formulas in pharmacies because magistral formulas would continue not to be regulated under EU law according to Article 1(5)(a) of the Commission’s proposal. Since this limitation of the scope of the Directive is a decision of the legislator, this limitation cannot be overruled by a non-legislative act within the meaning of Article 28 in conjunction with Annex VII of the Commission’s proposal. Furthermore, Article 28 does not confer any competence to change the scope of the (proposed) Directive either.

Phage therapeutics manufactured as magistral formulas in pharmacies would therefore continue to be regulated not under EU law but under the law of the respective Member State, so that harmonised standards for these individualised phage therapeutics would still not be possible at the EU level. The additional planned revision of Regulation (EC) No. 726/2004 will not change this either, because magistral formula medicinal products are not covered by the amended directive and thus, in turn, not covered by the amended regulation [140].

Article 28 of the proposal would therefore at best be relevant for patient-specific phage medicinal products (due to variable composition) that are manufactured by pharmaceutical entrepreneurs, i.e., not in a pharmacy. This could possibly overcome the translation problems of this manufacturing variant described in Section 5.6.

Whether the proposed Article 28 would have significance for individualised GM phages in the hospital exemption is questionable. Also, under the Commission’s proposal, certain GM-phages would continue to be GTMP and thus ATMP. In addition, the hospital exemption would still be granted under Article 2 of the Commission’s proposal. It would therefore first be necessary to clarify whether individual GM-phages are subject to the ATMP Regulation or to the rules adopted on the basis of Article 28 of the proposal.

Hence, as long as the EU and the Member States allow individualised phage therapeutics of the magistral formula not to be regulated by EU law, no harmonised regulatory framework for (GMP) quality, safety, efficacy, and marketing authorisation can be developed for such (phage) medicinal products at the EU level.

That at least a partially harmonised legal framework for the GMP standard, for traceability, and for pharmacovigilance is possible for individualised therapeutics is demonstrated by the legal framing of the hospital exemption for ATMP. If phage medicinal products are to be harmonised across the EU for the benefit of patients, then, firstly, pre-manufactured wild type phage medicinal products should be subject to a mandatory centralised EU authorization. This would be the case under the proposed revision of Regulation (EC) No. 726/2004 [140], either as “priority antimicrobial” or due to the novelty of the active substance. Secondly, a provision similar to the hospital exemption could be included in Directive 2001/83/EC or the revised Directive to require Member States to ensure that, for individualised phage medicinal products, national traceability and pharmacovigilance requirements and specific quality standards are equivalent to those that apply at the Community level for phage medicinal products.

Finally, contrary to the current wording of Directive 2001/83/EC and the wording in the EU Commission‘s proposal to reform Directive 2001/83/EC [44], it should be technically and legally unambiguous whether phage-based medicinal products are biological medicinal products (Section 4.11), so that the specific provisions for biological medicinal products can be applied to phage-based medicinal products in a legally watertight manner.

## Figures and Tables

**Table 1:** Legal classification of phages and further substance for phage therapy.

Technical/Scientific Classification	Legal Classification
Phages wild type, genetically modified, mutated, etc.	pharmaceutical substances, Art. 1(3) Directive 2001/83/EC *see* *Section 4.1* active substance, Art. 1(3a) Directive 2001/83/EC *see* *Section 4.2*
Pharmacologically inert auxiliary substances added to phages to form ready-to-use phage-based therapeutic	excipient, Art. 1(3b) Directive 2001/83/EC *see* *Section 4.2*
Instrument, apparatus, article, etc., used for the application of phage therapeutics	medical devices Art. 2 Regulation (EU) No. 2017/745 *see* *Section 4.2* *and Section 4.8*
Therapeutic (=active substance + excipient) containing wild type phages	simple medicinal product, Art. 1(2) Directive 2001/83/EC *see* *Section 4.12*
Therapeutic containing “trained” phages	simple medicinal product, Art. 1(2) Directive 2001/83/EC *see* *Section 4.9.3*
Therapeutic containing mutated phages (regardless of method used)	simple medicinal product, Art. 1(2) Directive 2001/83/EC *see* *Section 4.9.2*
Therapeutic containing genetically recombinant phages	gene therapy medicinal product, Annex I, Part IV, 2.1(1st sentence) Directive 2001/83/EC *see* *Section 4.10*
Genetically modified phages for vaccination by phage display	immunological medicinal product, Art. 1(4) Directive 2001/83/EC, not gene therapy medicinal product, Annex I, Part IV, 2.1(2nd sentence) Directive 2001/83/EC *see* *Section 4.6* *and Section 4.9.1*

**Table 2:** Legal basis for the different settings of phage therapy. Art. = Article, MA = marketing authorisation, n/a = not applicable, WTP = wild type phage, GTMP = gene therapy medicinal product (subcategory of ATMP = advanced therapy medicinal product according to Regulation (EG) No. 1394/2007), (**-**) = not possible; (**?**) = provided that certain legal requirements are met.

	Industrial Manufacturer Pre-Manufactured and Prepared on Demand *See* *Section 5.3* *and Section 5.6*	(Hospital) Pharmacy Prepared on Demand *Magistral Formula* *See* *Section 5.4*	Physician Made and Administered Prepared on Demand *See* *Section 5.5*
Phage amplification (WT, training, Recombinant) *see* *Section 5.2*	Art. 46(f) Directive 2001/83/EC Art. 46b Directive 2001/83/EC GMP standard	Directive 2001/83/EC **(n/a)**, Art. 3(1), therefore, no EU legal requirement for manufacturing licence or GMP standard. Thus, the respective provisions in the individual EU member states apply.	Directive 2001/83/EC **(n/a)**, Art. 2(1), not intended to be placed on the market Regulation (EC) 726/2004 **(n/a)**, Art. 3 not intended to be placed on the market merely EU Member State law, check whether a national authorisation is required.
Preparation medicinal product (WTP, training, Recombinant)	mandatory manufacturing license, Art. 40(1), 46(f) Directive 2001/83/EC GMP standard		
WTP medicinal product placing on the market	MA by EU Member State law **(-)** mandatory EU centralised MA Art. 3(1) Regulation (EC) 726/2004 **(?)** optional EU centralised MA Art. 3(2) Regulation (EC) 726/2004	**(-)** EU MA Technically speaking, the magistral formulas are placed on the market, but no MA is required under EU law, because according to Art. 6 of Directive 2001/83/EC, only medicinal products that fall within the scope of this Directive require MA. However, according to Art. 3(1) of Directive 2001/83/EC, magistral formulas are excluded from the Directive. Thus, provisions of the individual EU member states apply. *see* *Section 5.4.3* **(?)** MA by EU Member State law, check whether a national authorisation is required.	
Recombinant phages (=GTMP) placing on the market	**pre-manufactured** mandatory EU centralised MA Art. 3(1) Regulation (EC) 726/2004 **Prepared on demand** hospital exemption (=MA by EU Member State law) Art. 28 Regulation (EC) 1394/2007, Art. 3(7) Directive 2001/83/EC		
Legal consequences and effects *see* *Section 9*	Harmonised EU-wide regulation	No EU harmonised regulation	no EU harmonised regulation

## Data Availability

Data are contained within the article.
